# 2001. IDWeek Oral Abstract Coaching Pilot Program

**DOI:** 10.1093/ofid/ofad500.128

**Published:** 2023-11-27

**Authors:** Dana Blyth, James B Cutrell, Erin Bonura, Vera Luther, Michael T Melia

**Affiliations:** Walter Reed National Military Medical Center, USU, Bethesda, MD; UT Southwestern, Dallas, TX; Oregon Health & Science University, Portland, Oregon; Wake Forest University School of Medicine, Winston Salem, NC; Johns Hopkins University, Baltimore, MD

## Abstract

**Background:**

Oral abstract presentations (OAP) at national meetings are high visibility opportunities. However, there has not been a formal process for coaching presenters and providing feedback at IDWeek. We led a pilot project pairing ID-trained medical educators with OA presenters for feedback prior to and at IDWeek.

**Methods:**

A link in IDWeek 2022 OAP acceptances invited presenters to participate in the OAP coaching pilot program (CPP). The link requested demographics, availability of local mentors to provide feedback, and areas of desired feedback for the OAP. Coaches were primarily solicited from the IDSA Med Ed Community of Practice Workgroups to give feedback pre-IDWeek, attend the IDWeek OAP, and provide feedback after. An anonymous survey was sent to presenters and coaches 8 days post-IDWeek.

**Results:**

38 presenters (median of 1 (IQR 0, 4) prior national OAP) requested to participate, despite 71% already having feedback available (table 1). Due to coaching pool limitations, presenters with no prior national OAPs and/or without local feedback were prioritized for the CPP. 22 (85%) and 18 (69%) of the 26 coaches and presenters responded to the post-IDWeek survey, respectively (table 2). Presenters and coaches largely overlapped regarding the types of feedback requested and delivered before and at IDWeek (table 3). Presenter qualitative comments highlighted the value provided in improving OAP visual display, delivery, clarity for those unfamiliar with the work, and the presenter’s confidence. Coaches described appreciation for the opportunity to practice providing feedback and feelings of pride and fulfillment in the improvements noted following the feedback. Both presenters and coaches commented on the benefit of meeting new colleagues and building networks. 94% of presenters and 91% of coaches reported professional/personal benefit from the CPP.

**Table 1:**
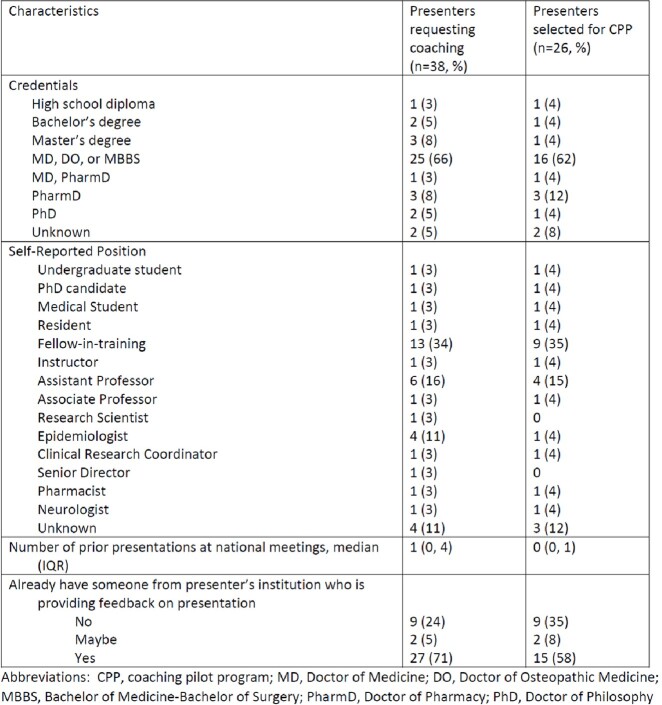
Characteristics of presenters requesting and selected to participate in the IDWeek Oral Abstract Coaching Pilot Program

**Table 2:**
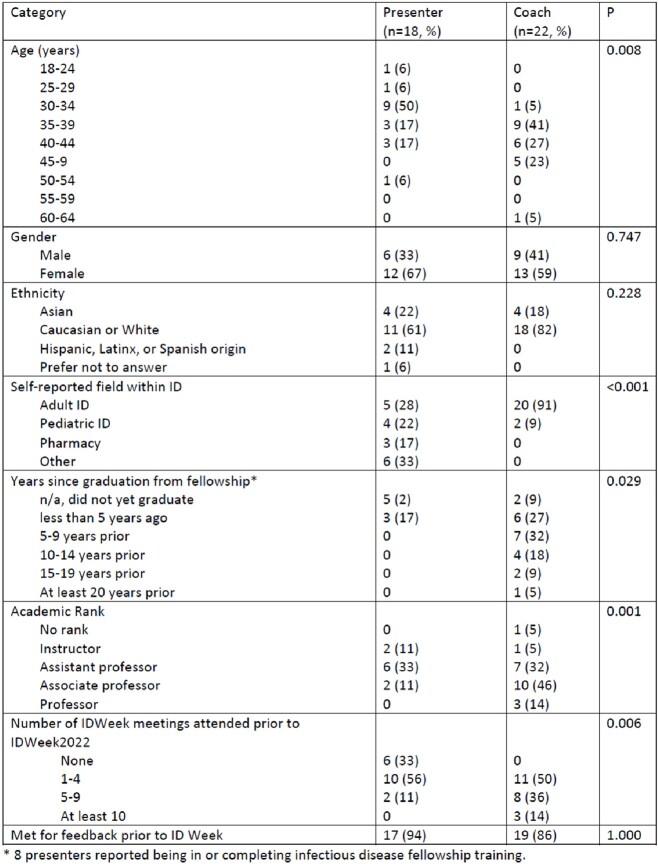
Demographics of presenters and coaches in the IDWeek Oral Abstract Coaching Pilot Program

**Table 3:**
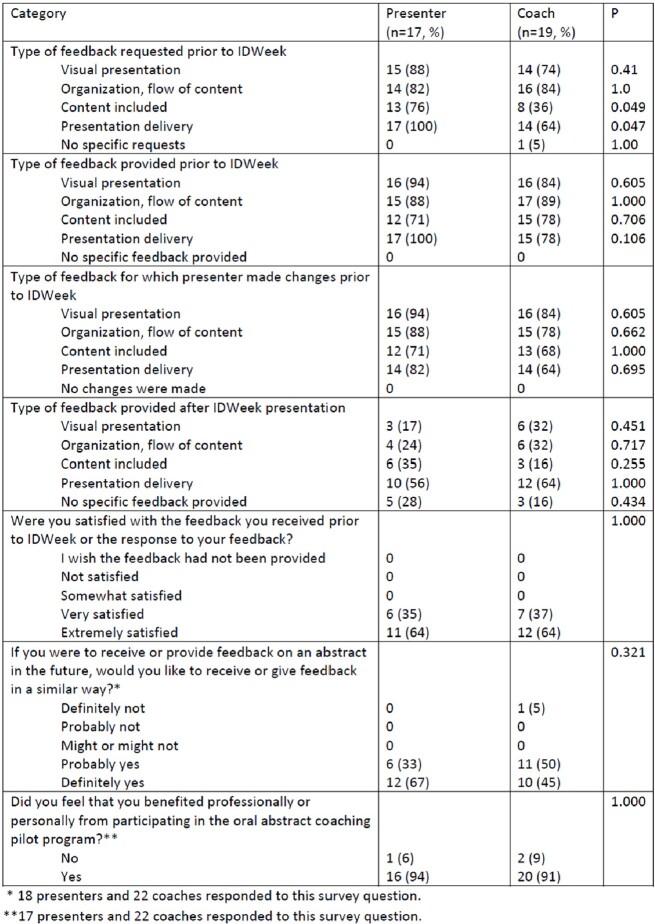
Results of IDWeek Oral Abstract Coaching Pilot Program evaluation survey

**Conclusion:**

This OAP CPP was highly successful, with > 90% of presenters and coaches reporting professional/personal benefit and interest in participating again. In the future, a coach’s guide will be incorporated to increase feedback effectiveness across the desired OAP topics and to augment expansion of the pool of potential coaches. We hope coaching targeting these early career presenters can enhance engagement and recruitment into ID.

**Disclosures:**

**James B. Cutrell, MD**, IDSA: stipend as Deputy Editor for Open Forum Infectious Diseases

